# Development and evaluation of a cultural competency training curriculum

**DOI:** 10.1186/1472-6920-6-38

**Published:** 2006-07-26

**Authors:** David H Thom, Miguel D Tirado, Tommy L Woon, Melen R McBride

**Affiliations:** 1 Department of Family and Community Medicine, University of California, San Francisco, San Francisco General Hospital, 1001 Potrero Avenue, Building 80/83, San Francisco, CA 94110, USA; 2 California State University, Monterey Bay, 100 Campus Center, Seaside, CA 93955, USA; 3 Dartmouth College, 6217 Collis Center, Room 211G, Hanover, NH 03755, USA; 4 Stanford Geriatric Education Center, School of Medicine, Stanford University, c/o VAPAHCS, 3801 Miranda Avenue, Building 4, 3^rd ^Floor, Palo Alto, CA 94304, USA

## Abstract

**Background:**

Increasing the cultural competence of physicians and other health care providers has been suggested as one mechanism for reducing health disparities by improving the quality of care across racial/ethnic groups. While cultural competency training for physicians is increasingly promoted, relatively few studies evaluating the impact of training have been published.

**Methods:**

We recruited 53 primary care physicians at 4 diverse practice sites and enrolled 429 of their patients with diabetes and/or hypertension. Patients completed a baseline survey which included a measure of physician culturally competent behaviors. Cultural competency training was then provided to physicians at 2 of the sites. At all 4 sites, physicians received feedback in the form of their aggregated cultural competency scores compared to the aggregated scores from other physicians in the practice. The primary outcome at 6 months was change in the Patient-Reported Physician Cultural Competence (PRPCC) score; secondary outcomes were changes in patient trust, satisfaction, weight, systolic blood pressure, and glycosylated hemoglobin. Multiple analysis of variance was used to control for differences patient characteristics and baseline levels of the outcome measure between groups.

**Results:**

Patients had a mean of 2.8 + 2.2 visits to the study physician during the study period. Changes in all outcomes were similar in the "Training + Feedback" group compared to the "Feedback Only" group (PRPCC: 3.7 vs.1.8; trust: -0.7 vs. -0.2 ; satisfaction: 1.9 vs. 2.5; weight: -2.5 lbs vs. -0.7 lbs; systolic blood pressure: 1.7 mm Hg vs. 0.1 mm Hg; glycosylated hemoglobin 0.02% vs. 0.07%; p = NS for all).

**Conclusion:**

The lack of measurable impact of physician training on patient-reported and disease-specific outcomes in the current has several possible explanations, including the relatively limited nature of the intervention. We hope that the current study will help provide a basis for future studies, using more intensive interventions with different provider groups.

## Background

Health disparities in the United States between European-Americans and other racial/ethnic groups have been amply documented [1]. Increasing the cultural competency of physicians and other health care providers has been suggested as one mechanism for reducing such disparities by improving the quality of care across racial/ethnic groups [1-5]. The Federal Office of Minority Health has published guidelines that include cultural competency training for health care providers [6] and the Accreditation Council on Graduate Medical Education has made cultural sensitivity a part of professional competency for physicians in training [7].

Cultural competency training is increasingly common in medical school [8,9], and as part of residency training [10]. While over a dozen studies have evaluated the effect of such training on the skills, attitude, knowledge and performance of medical students [11], few studies have evaluated training curricula for residents or physicians in practice. One study evaluated a cross-cultural training curriculum in residents [10] by comparing resident self-assessed cultural competency at the beginning of their second year and end of third year. Most studies have used physician self-assessment and post-training examination as the outcome measure, although a few have also included observed physician behaviors [11,12]. We could locate only one study which assessed a patient outcome (satisfaction) before and after physician training (a Spanish language immersion course) [13]. No study was found which used patient reports of physician behaviors or which evaluated the impact of training on patient health outcomes.

To help advance the field of cross-cultural training for physicians, we sought to develop and evaluate a brief cross-cultural curriculum for resident and practicing physicians based on a model of culturally competent physician behaviors, and to evaluate the training plus feedback compared to feedback alone with respect to changes in patient-reported physician behaviors, patient satisfaction, patient trust in his or her physician, and disease-specific patient health outcomes.

## Methods

### Study design

**Figure 1:**
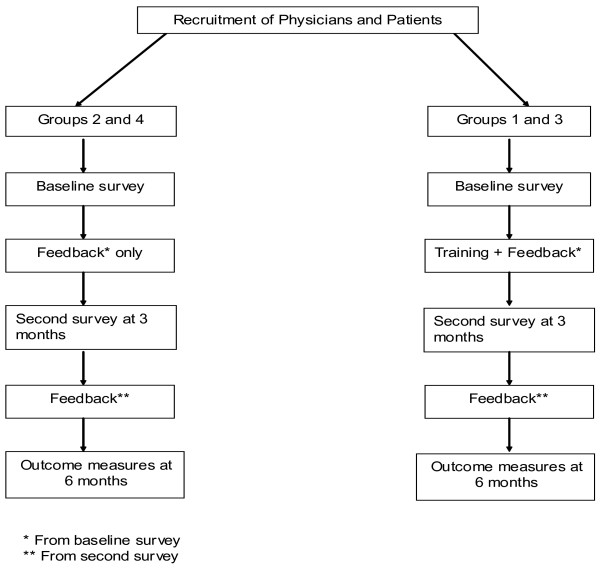
Flowchart showing recruitment, intervention and outcomeassessment. * From baseline survey. ** From second survey

The structure of the study is provided in figure 1 and described in detail below. Baseline measures were obtained from 4 practice sites in northern California. Physicians at all 4 sites received feedback on cultural competency behaviors reported by their patients. Two practice sites (sites 1 and 3) were randomly assigned to also receive the training intervention. Changes in outcome measures for patients in the "Feedback + Training" sites (1 and 3) were compared to "Feedback Only' sites (2 and 4), controlling for differences in baseline patient and physician characteristics. The schedule for providing training, feedback, and data collection points are summarized in Figure 1. Physicians were not randomized to the training intervention because of concern about creating a 'halo effect,' where trained physicians could transmit some of the training effect to their untrained colleagues working in the same clinic.

### Setting and subjects

The study was conducted at 4 locations: an academic, medical center-based family practice (Site 1), a community-based primary care practice (Site 2); a rural family medicine residency program (Site 3), and an inner-city family medicine residency program (Site 4). Physicians were recruited at all sites by one of the study investigators (DT). Patients of these physicians who had been seen in the past 12 months and had at least one visit for diabetes (ICD 9 code 250) or hypertension (ICD 0 code 401 to 405) were identified from computerized billing and encounter records. Enrolling patients with diabetes or hypertension allowed us to use the disease specific outcomes of weight loss, and control of blood pressure and glucose levels. Patients were recruited for the study via mail, with a follow-up phone call if needed after the second mailing. The recruitment letter and screening questionnaire were mailed in English and Spanish to patients identified as Hispanic or probably Hispanic (from self-designation in the computerized records, when available, or by surname), and in English and Chinese to patients identified as Asian or probably Asian (by computerized records or surname). Questionnaires were administered and returned by mail except for participants who requested to complete the questionnaire by phone. Participating patients were paid $10 for returning the baseline questionnaire, and $5 for each follow-up questionnaire. Follow-up questionnaires were sent 2 times. The study was approved by institutional review boards (IRBs) at Stanford University and at the University of California, San Francisco.

### Baseline and post-intervention measures

The primary outcome measure used was the Patient Reported Physician Cultural Competency (PRPCC) Scale, which asked patients of physicians in the study to report physician behaviors previously identified as being important for cultural competency [14]. Briefly, the PRPCC asks patients to report the frequency their physician exhibited each of 13 behaviors, listed in Table 1, (1 = never, 2 = seldom, 3 = sometimes, 4 = usually, 5 = always). The scale score was transformed to a 0 to 100 scale by taking the total score (TS), dividing by 13 to get a mean score, subtracting 1, then multiplying by 25: ([TS/13]-1) × 25 = 0 to 100. The physician behaviors assessed by the PRPCC were derived from the same general model of cultural competency from which the training curriculum for the study was developed.

Secondary outcome measures were patient satisfaction, patient trust in the physician, and disease specific outcomes. Specifically, patient satisfaction with their physician was assessed using 10 items from the Patient Satisfaction Questionnaire [15] (sample item: rating doctor with regard to "...respect and courtesy shown to me"). Patient trust was measured using the 7 likert-response items in the trust subscale of the Primary Care Assessment Survey [16] (sample item: "I completely trust my doctor's judgments about my medical care"). Like the PRPCC scale, both satisfaction and trust scores were transformed to 0 to 100 scale. Patient medical records were abstracted to obtain outcomes of care: change in weight, change in blood pressure (if hypertensive), and change in glycosylated hemoglobin (if diabetic).

### Curriculum content and teaching methods

Our model of cultural competent primary health care was adapted from a model developed by one of the co-authors, Dr. Miguel Tirado, under a grant from the Health Services and Research Administration (HSRA) of the U.S. Department of Health and Human Services. The model was based on the results of two panels of Latino and Chinese physicians serving minority patients convened to provide expert opinions on how to deliver more culturally competent care to patients with language and cultural differences from their providers. While working separately, these panels developed comparable sets of competencies which were grouped into 3 categories: knowledge of patients (including knowledge of patients cultural health beliefs and identification of their level of acculturation with respect to mainstream health beliefs); communication skills (including listening, explaining, acknowledging, providing recommendations, and working effectively with interpreters); and cultural brokering (including negotiating a treatment plan with patient and family, understanding community resources available to patients, and working with then healthcare system to meet the needs of culturally diverse patients). Within this framework, the training curriculum content was developed based on a review of published curricula [10,17], studies of cultural competency [2,18], ethnogeriatric curricula at Stanford Geriatric Education Center, as well as the experiences of the authors in teaching cross-cultural competency. The curriculum was field tested with 18 primary care physicians and was refined based on participant feedback.

We elected to divide our training into 3 modules corresponding to each of the 3 areas in our cultural competency model. Modules could then be given either as a single half-day training session or as 3 separate sessions lasting 1 to 1.5 hours each. While there was a focus on patients with diabetes or hypertension, most of the content was designed to be applied to patients in general. Teaching techniques included didactic presentations, group discussion, role-playing with learners critique, group exercises, use of trigger tapes, and handouts. Instructors included the authors of this paper, two other physicians with expertise in cross-cultural care, and experts in training and use of interpreters. The concept of a cultural competency continuum was used across all 3 modules to emphasize working toward a goal of increasing cultural versatility, defined as "having a variety of skills to bridge to patients from different cultural backgrounds."

The objectives for Module 1, "Expanding Knowledge of Ethnic Patients," were (1) to discuss the cultural gap between provider's and patient's knowledge and belief systems, (2) present information about the incidence, prevalence and complications of diabetes and hypertension in different racial/ethic groups, (3) provide examples of culturally-based beliefs and practices, and (4) to teach techniques for assessing beliefs and practices of individual patients. Epidemiologic data on diabetes and hypertension among Latino, Asian, White, and African-American patients was presented in an interactive didactic format using slides and handouts. Learners were asked to share their knowledge of and experience with cultural beliefs related to diabetes and hypertension; instructors then provided examples of common cultural beliefs and practices in each of the target populations. Instructors role-played an encounter between a doctor and a Latino patient with newly diagnosed diabetes which was critiqued by the learners. The encounter was used to illustrate the need to identify gaps in understanding and to suggest techniques for increasing understanding to fill these gaps.

Objectives for Module 2, "Enhancing Communication Skills for Cultural Competency," were (1) to present techniques for eliciting the patient's explanatory disease model [19,20] and use of traditional treatments, (2) to apply the LEARN model [21] to the patient interview and (3) to model problematic and improved physician communication. Specific questions were provided that could be used to elicit the patient's explanatory model of their illness such as "What do you think caused this problem?" Examples were also given of the application to the patient interview of the LEARN mnemonic (**L**isten sympathetically to the patient's perception of the problem, **E**xplain your perceptions of the problem, **A**cknowledge and discuss differences and similarities, **R**ecommend a treatment plan, **N**egotiate agreement). A videotape was presented of a problematic interaction between a young female Asian-American physician and an older, traditional Asian man with hypertension and kidney disease, followed by a group discussion about how the interview could be improved by application of the above techniques. A second, 'improved' version of the same encounter which included some of these techniques was then shown and discussed.

Module 3, "Use of Interpreters and Cultural Brokering," had as its objectives (1) understanding the importance of working with trained interpreters and how to use interpreters effectively, (2) negotiating a treatment plan with the patient and family and (3) filling the role of a cultural broker by connecting the patient to community and health plan resources. This module began with a memory exercise to show the challenge interpreters face in conveying information accurately. We then described the roles, responsibilities, and different types of interpreters and gave examples of less effective and more effective ways to work with an interpreter. Role playing was used to provide experience with use of an interpreter. A video tape was shown which demonstrated both common pitfalls in interpretation and model physician behaviors to increase the effectiveness of the interpreter. Training materials are available by request from the first author (DT).

Post-training, learners were asked to rate the usefulness of the training they received on a 5 point Likert-type scale, with 5 being 'excellent' and 4 being 'very good'. Learners' ratings for 10 specific areas of training ranged from 4.0 to 4.5 with a mean of 4.31. In addition, learners rated the overall educational content as a 4.30 (mean score), relevance to practice as 4.55 (mean) and reported that the training had increased their awareness of patient's cultural beliefs (mean = 4.30) and increased their communication skills with patients (mean = 4.45).

### Feedback

Feedback was provided to each physician via written report with an interpretation of the aggregated PRPCC scores from their patients. In addition to an overall score, scores were provided for each of the 3 areas: history taking, explaining, and partnering. Scores for each individual item were reported as well. Aggregated mean scores for other physicians within their practice group were provided for comparison. A cover letter accompanying the feedback offered interpretation of the scores and suggested behaviors that could help to improve scores. The physician was offered the opportunity to discuss one-on-one the feedback results. No information was provided which could be used to identify individual patients or other physicians.

### Analytic approach and statistical methods

Sample size calculations showed that 175 participants in each group would provide a power of .80 to detect a significant (p < .05) difference in PRPCC scores between the two groups if the true difference was at least 0.3 standard deviations. Baseline characteristics for the "Feedback Only" and the "Training + Feedback" groups were compared using Chi-square test for dichotomous or categorical variables and Student's t-test for continuous variables. Differences in outcomes were analyzed only for the participants who had one or more visits to the study physician during the study period. All outcome variables exhibited a near normal distribution pattern. Mean changes in patient outcome measures from baseline to end of the study were calculated for each group and compared between groups using multiple analysis of variance (MANOVA) to adjust for differences in patient and physician characteristics and levels of each outcome variable at baseline. Analyses were done in SPSS version 13.0.

## Results

### Subject recruitment and enrollment

A total of 86 physicians were contacted of which 53 (62%) agreed to participate in the study. The mean age of participating physicians was 39.2 years and 24 (45%) were female. Thirty-three (62%) were family physicians in practice and 20 (38%) were family medicine residents. Thirty-eight (72%) were White, 8 Latino, 5 Asian-American, 1 African-American and 2 other. Forty-three spoke another language (in addition to English), of which 34 spoke Spanish. Compared to participating physicians, the 23 non-participating physicians were younger (mean age = 35.7 years) and more likely to be residents (61%). Race/ethnicity and language abilities were not assessed in non-participating physicians.

Approach letters were sent to a total of 2837 patients, of which 940 had moved out of the area or could not be contacted. Of the 1897 patients contacted or whose status could be determined, 483 did not identify the study physician as their primary care physician, 186 did not have a diagnosis of either diabetes or hypertension, and 9 had died. Of the 1219 potentially eligible patients contacted, 671 (55.0%) agreed to participate and were enrolled. Of the 671 enrolled patients, 429 (63.9%) completed the first survey and 320 completed all surveys for a completed response rate of 26.2% of potentially eligible patients (320/1219).

Non-respondents were compared to respondents with respect to key variables of age, gender, race, diagnosis, and primary language. There were no significant differences between non-respondents and respondents with respect to the following variables: mean number of visits to their physician prior to the study (3.8 vs 3.7) or during the study (2.7 vs. 2.9), mean age (60.1 vs. 59.4 years) and percent female (54 % vs 60%). However, non-respondents were substantially more likely to be Asian (40% vs. 18%, p < .001) and not primary English speakers (54% vs 32%).

There were no significant differences in physician gender or training status in the "Feedback Only" and "Training + Feedback" groups, as shown in Table 2. While physician race/ethnicity as a categorical variable did not differ significantly between the groups, there was a substantially greater proportion of Latino physicians in the "Training + Feedback" group.

Patient demographics, diagnoses, length of relationship and number of visits with physician, and values for outcome variables measured at baseline are shown in Table 3. Compared to patients in the "Feedback Only" group, patients in the "Training + Feedback" group were significantly younger and less likely to be female, to have a primary language other than English to be born outside the United States, and to self-identify as Latino or Asian. Patients in the "Training + Feedback" were more likely to have hypertension and less likely to have diabetes only, and had fewer visits to their physician before and during the study. Baseline levels of patient-reported physician cultural competence, patient trust and patient satisfaction were similar between the two groups. However, patients in the "Training + Feedback" group were slightly heavier and on the average, had better controlled blood pressure and blood glucose levels.

As shown in Table 4, there was relatively little change in the outcome variables in either intervention group. There were no significant group differences in the amount of change for any outcome variable after controlling for differences in patient characteristics and baseline values of the outcome measures. To investigate if this apparent lack of effect was limited to patients with fewer visits during the study, we repeated these analyses for patients with less then 3 visits during the study period and those with 3 or more visits. There were no significant differences in outcomes by intervention in either group. Additional, post-hoc analyses were conducted to look for changes by patient subgroups, including patients whose primary language was or was not English, older and younger patients, male and female patients, and patients of monolingual vs. bilingual physicians. There were no significant differences between intervention groups for any subgroup. In addition, there was no significant difference in changes of any outcome among the 4 sites.

## Discussion

We did not find any measurable impact of a brief (4.5 hours) training curriculum aimed at improving physician cross-cultural knowledge and skills on any of the outcomes we chose. While other cultural competency studies have shown positive training effects on physician knowledge, attitudes and skills (by physician self-report) [11,12], no previous study has apparently reported changes in patient-centered outcomes and disease-specific outcomes measured over a period of 6 months.

The lack of a statistically significant effect of training in the current study is not entirely surprising. Studies that have reported an effect of training on other physician behaviors have used more extensive training, including a two-week Spanish immersion course [22] and a multi-session training with practice time between sessions [23]. Even with these more intensive training programs, changes in physician behavior were relatively modest and were not demonstrated to persist beyond a few months. Typical continuing medical education programs have been shown to have limited utility in effecting changes in physician behaviors [24,25]. The value of web-based programs has not been well studied. Sustained changes in physician behaviors may require a combination of interactive training, dedicated practice time, and reinforcement of behavioral changes in the practice environment. We did not find any studies of combined physician training and delivery system changes for improving physician cultural competency. Regardless of the type of training, it is important to evaluate the effect of the training on physician behaviors and, if possible, patient outcomes.

A recent systematic review of the methodological quality of studies evaluating cultural competence training of health professionals [12] concluded that "most did not adhere to basic principles of study design, reporting and data analysis." Of 64 studies evaluated "only eight had adequate comparison groups." A strength of our study was the use of a concurrent control group design to detect secular and other changes not related to training or feedback. The intervention and primary outcome measure were both developed from the same conceptual model to provide concordance between skills taught in the second training module and the behaviors measured. Module 1 focused on increasing physician knowledge as a preparation for the behavioral changes taught in module 2. Module 3 was devoted to using of interpreters – an important dimension of cultural competency but one that can only be assessed for non-English proficient patients. Because less then a third of our participants reported a primary language other than English, we did not have the power to assess behavioral changes in interpreter use.

Our study had several important limitations. Chief among these is the relative brevity of the training and the lack of institutional-based support and reinforcement for changing behaviors. The ability of changes in physician behavior to improve clinical outcomes was also limited by the number of post-training visits to the study physician. In addition, physicians at our training sites, while not formally trained in cross-cultural care prior to the study, may have been relatively experienced by virtue of serving a diverse patient population. For example, over 70% of patient participants were from racial/ethnic groups other then Caucasian, non-Hispanic. Furthermore, 34 of the 53 participating physicians (64%) spoke Spanish (though we did not ask them to self-assess for fluency), compared to just 26% of primary care physicians in a recent study using mail survey [26]. By choosing to provide feedback to both the training and the comparison groups, we may have biased our study against finding an effect of the training. Our study was powered to find a significant difference if the true difference in outcomes was at least 0.3 standard deviations; thus smaller, though still clinically important differences could have been missed. Effects limited to a subgroup, such as non-English speaking patients seeing monolingual physicians, could not be adequately evaluated because of limited sample size. It is possible that non-respondents, which were more likely to have low English proficiency and to be Asian, might have reported physician behaviors differently. However, within the set of participating patients, we found no evidence that Asian or low English proficient patients were more likely to report a change in physician behaviors then other patients. Finally, we asked the patient participants to report on their physicians' behaviors in general. It is likely that even if physician behaviors were changed by the intervention, patients may average behaviors over several visits, including visits prior to the intervention, thus reducing our ability to show a difference. In future studies of this type, it would be preferable to ask about behaviors with respect to specific visits before and after an intervention.

## Conclusion

As has been noted by Betancourt [4], the pathway from training providers in cross-cultural care to improving patient health outcomes is a long one, and traversing the path requires successfully connecting training to changes in behavior, and changes in behavior to improved patient outcomes. Our primary goal in the current study, to effect a significant measurable change in patient-perceived physician behaviors, was not achieved. It is likely that a stronger intervention – with a longer period of training and practice time and regular reinforcement over time – is needed to effect a measurable behavioral change. Effecting changes via physician training on patient trust, satisfaction, or 'hard' disease-specific outcomes is an even higher bar to reach. While we found no training effect on patient trust, satisfaction, or disease-specific outcomes, this should not be interpreted as indicating that cultural competency training is necessarily without value. Rather, the value of cultural competency training remains to be established. We also believe that including clinically relevant outcomes measures in an intervention study is worthwhile for at least 2 reasons: (1) behavioral changes alone are unlikely to be valued unless they can be tied to clinical outcomes and (2) physician training may affect outcomes in ways not mediated by changes in patient-reported physician behaviors. W e hope that the current study, while not able to demonstrate an impact of physician training, will help provide a basis for future studies in this area.

## Competing interests

The Author(s) declare that they have no competing interests.

## Authors' contributions

DT was the principal investigator of the study, helped with development of training curriculum, supervised the data collection and analysis, and was the principal author for the paper. MT was co-principal investigator for the study, was primarily responsible for development of the cultural competency measure used, assisted with data interpretation and has participated in the writing of the paper. MM and TW helped develop the training curriculum, participated in the training intervention, and reviewed and improved this paper.

## Pre-publication history

The pre-publication history for this paper can be accessed here:



## Figures and Tables

**Table 1:** Patient Reported Provider Cultural Competency (PRPCC) Scale Items With 5-Point Rating Scale (1 = never, 2 = seldom, 3 = sometime, 4 = usually, 5 = always)

1. My doctor asks me why I think I got sick.
2. My doctor talks with me about medications I may use other than the ones he/she prescribes.
3. My doctor talks with me about traditional healing remedies I may use.
4. My doctor asks if I seek advice from other family members and friends in making decisions about my health care.
5. When discussing diagnosis and treatment related to my condition, my doctor asks if I would like to include family members in the discussion.
6. My doctor takes time to help me understand possible side effects of the medications he or she prescribes for me.
7. My doctor informs me of the resources in my local community where I can find help.
8. My doctor asks if I understand his/her instructions and if not repeats them when necessary.
9. My doctor asks if I have other questions or concerns before I leave the office.
10. My doctor helps me to ask questions about my condition and treatment.
11. My doctor helps me answer the questions he or she asks.
12. My doctor encourages me to stop him or her when I am confused.
13. My doctor helps me make decisions about my treatment.

**Table 2:** Comparison of characteristics of participating physicians (percent) by intervention group

**Characteristic**	**Training + Feedback (n = 23)**	**Feedback only (n = 30)**	**Chi Square and P-value**
Female	41%	52%	NS
Resident physician	50%	61%	NS
Race			NS
Caucasian	64%	80%	
Latino	27%	7%	
African-American	0%	3%	
Asian-American	9%	10%	

**Table 3:** Comparison of characteristics of participating patients by intervention group (percent or mean ± s.d.)

**Characteristic**	**Training + Feedback (n = 247)**	**Feedback only (n = 182)**	**P-value**
Demographic Characteristics			
Age	54.9 ± 11.6	62.1 ± 11.4	<0.01
Female	48.8%	63.2%	<0.01
Less than high school education	27.0%	31.3%	NS
Primary language other than English	14.4%	45.1%	<0.01
Born outside United States	25.8%	53.6%	<0.01
Race/ethnicity			<0.05
Caucasian non-Hispanic	30.0	21.9%	
Latino/Hispanic	25.3	28.9%	
African-American	23.2	20.3%	
Asian	13.1	23.0%	
Other	8.4	5.9%	
Diagnosis			<0.01
Diabetes only	16.9%	5.8%	
Hypertension only	57.2%	55.6%	
Hypertension and diabetes	25.8%	38.6%	
Relationship with physician			
Length of relationship			<0.05
Less than 1 year	34.6%	24.5%	
1 to 4 years	44.0%	44.1%	
More than 4 years	21.4%	31.3%	
Number of visits in previous 6 months	2.8 ± 2.1	3.3 ± 2.6	<0.05
Number of visits during study	2.5 ± 2.1	3.2 ± 2.3	<0.01
Outcome Measures			
Cultural competency	52.4 ± 21.6	54.5 ± 24.1	NS
Trust	79.9± 15.1	78.4 ± 15.4	NS
Satisfaction	71.0 ± 23.3	69.6 ± 22.1	NS
Weight	191.7 ± 51.3	183.3 ± 51.6	NS
Blood pressure (systolic) +	134.6 ± 15.2	137.3 ± 18.6	.<0.05
Glycosylated hemoglobin++	7.4 ± 1.5	8.1 ± 2.1	<0.05

+ patients with hypertension only.

++ patients with diabetes only

**Table 4:** Comparison of changes in outcomes by type of intervention status for participating patients with 1 or more office visit during the study (mean ± s.d.)

Outcome	Training + feedback (n = 23)	Feedback only (n = 30)	Manova and p-value*
PRPCC score	3.66 ± 17.2	1.78 ± 19.2	NS
Satisfaction score	-0.73 ± 17.6	-0.21 ± 17.8	NS
Trust score	1.93 ± 8.6	2.54 ± 15.6	NS
Weight (pounds)	-2.46 ± 17.7	-0.66 ± 9.4	NS
Systolic blood pressure (mm hg)+	1.69 ± 17.0	0.07 ± 15.6	NS
Hemoglobin A1C (%)++	0.02 ± 0.8	0.07 ± 1.69	NS

* Adjusted by MANOVA for patient age, gender, education, language, place of birth, race, diagnosis, length of relationship with physician, number of visit in 6 months prior to baseline, number of visits during study, baseline value of outcome variable, and physician race/ethnicity.

+ patients with hypertension only.

++ patients with diabetes only
